# The framework for analyzing the mechanism of the evolution of inter-city relationship networks on regional economic resilience

**DOI:** 10.1371/journal.pone.0308280

**Published:** 2024-10-04

**Authors:** Weihua Shi, Qi Wang

**Affiliations:** 1 Jilin University of Finance and Economics, Changchun, China; 2 Changchun University of Technology, Changchun, China; Al Mansour University College-Baghdad-Iraq, IRAQ

## Abstract

In this paper, we applied an analytical framework called "city cooperation intention → city relationship network → regional economic resilience," while considering the concept of externality as a measure of city cooperation intention, to draw insights from the perspectives of city relationships, biological evolution, and evolutionary economy. The evaluation system we developed focuses on the impact of inherent city culture on the inter-city relationship network, using variables such as knowledge spillover effect, technology symbiosis index, and market structure. We also incorporated innovation, resilience, and regeneration as determinants of regional economic resilience, building upon previous research findings. By applying structural force theory and a three-dimensional coordinate method, we analyzed the correlation between the relationship network and regional economic resilience, established a model to illustrate how the relationship network influences regional economic resilience, and described the course of action taken by the "three factors" of the relationship network on regional economic resilience. Ultimately, the aim of this study is to uncover the mechanism through which the inter-city relationship network affects regional economic resilience, with implications for healthy city design.

## 1. Introduction

In the era of globalization, there is a restructuring of internal connections among cities and regions. The synergies between natural resources, renewable resources, resource transformation, economic growth, urbanization, and carbon emissions continue to change [1, 2]. This has led to the gradual formation of a collaborative network between cities, with large cities at the core and encompassing small and medium-sized cities. The boundaries between renewable and non-renewable energy use and carbon emissions are being redrawn [3]. This transformation represents a shift from the traditional inter-city model to a network cooperation approach. Urban agglomeration emerges as a spatial organizational form of cooperation, where cities of different levels come together in a vast, multi-core, and multi-level consortium that reflects the division of labor and cooperation among them. The strength of economic resilience serves as a measure of the level of inter-city cooperation. Economic resilience is an inherent characteristic of urban agglomerations and a crucial attribute that enhances the long-term effective functioning of the economic system in cities and their surrounding regions [4].

Sustainable policies on energy efficiency, industrialization and environmental regulations can promote the formation of collaborative networks for sustainable development [5]. The establishment of an inter-city relationship network under the network cooperation model is a significant research topic for the recovery and enhancement of economic resilience in urban agglomerations. Understanding how the relationship network influences the economic resilience of urban agglomerations is a crucial scientific question within the theory of resilience recovery for such regions. Currently, most research on the economic resilience of urban agglomerations, whether in geography or economics, relies on macro-economic models and adopts an equilibrium analysis approach, which contradicts the principles of evolutionary economic geography. In contrast, research on inter-city relationship networks primarily relies on case analysis based on the theories and concepts of evolutionary economic geography [4]. It establishes a model that demonstrates the effects of inter-city relationship networks on the economic resilience of urban agglomerations, thereby uncovering the mechanisms through which these networks influence economic resilience. Such research is pivotal for understanding the mechanisms behind changes in the economic resilience of urban agglomerations.

Sustainable development while reducing carbon emissions is the key to promoting green economic development and enhancing regional economic resilience [6]. Amin et al. 2020 showed that the interaction between financial growth, environmental governance and environmental quality promoted the reduction of carbon emissions and sustainable economic development in the Middle East and North African countries, which in turn had a significant and direct impact on the deployment of renewable energy [7]. Li et al. 2024 [8] and Amin et al. 2022 [9] used the dynamic autoregressive distributed lag model to demonstrate the carbon emissions in China from 1996 to 2020. The results showed that indicators such as governance, trade, financial development, and renewable energy consumption have an adverse impact on carbon dioxide emissions, while urbanization and foreign direct investment have aggravated environmental degradation, and financial development and institutional quality help reduce carbon emissions and promote sustainable development. Amin et al. 2021 [10] argued that environmental degradation in developing economies such as Pakistan stems from competition among power plant owners, ambiguous markets, production procedures and lack of environment-related strategies, while the cost of power generation technology significantly affects production efficiency, production cost reduction, improvement of environmental conditions and sustainable development.

The primary cause for the stagnation and lack of growth of a regional economy can be attributed to the regional lock-in resulting from weak regional economic resilience. According to Glaeser [11], certain old industrial areas like Detroit suffer from a closed culture that hinders their willingness to collaborate with other cities in terms of labor division. This lack of cooperation prevents the city from effectively adapting to globalization [12]. Consequently, these regions experience talent shortages, a lack of innovation, reliance on a single industry, and ultimately become locked into their current state. In Northeast China, an established industrial base, the dominance of large state-owned heavy industries greatly outweighs the interaction with smaller external enterprises. As a result, there is a deficiency in technology, knowledge, and economic spillover. The cities in Northeast China primarily revolve around heavy industry and resource-based sectors, exhibiting the characteristics of being a single dominant city, thereby lacking the capacity for cross-border exploration of technical knowledge. This absence of an inter-city network focused on collaborative innovation further perpetuates the regional lock-in problem. The inter-city relationship network refers to the interactions and connections between cities influenced by their respective cultures [13]. This network affects various aspects such as resource allocation ability, innovation activities, division of labor, and specialization within urban agglomerations. The willingness to cooperate plays a significant role in establishing these relationships. Whether urban cultures are open or closed determines the intention of cities to cooperate and establish a relationship network. The cooperative intentions of cities are essential for their ability to adapt to globalization and have both positive and negative impacts on the economic resilience of urban agglomerations [14]. The willingness to cooperate reflects the inherent culture, knowledge, and systems of cities. For example, government will can be reflected in the establishment of consistency in economic and environmental policies to reduce environmental pollution, so as to achieve environmental sustainability [15], thereby enhancing environmental resilience [16]. The willingness to cooperate also indicates changes in their external tendencies regarding knowledge, culture, and customs. Further research is needed to understand the effects of cooperative intentions on inter-city relationship networks and their influence on the recovery and improvement of economic resilience in urban agglomerations. Enhancing the willingness to cooperate, promoting the establishment of inter-city relationship networks, and fostering the recovery and improvement of economic resilience in urban agglomerations are effective strategies to overcome economic challenges in Northeast China. Therefore, by analyzing the framework of "city cooperation intention → city relationship network → city group economic resilience," we can establish a quantitative analysis model to understand the influence of city group relationship networks on economic resilience. This analysis helps uncover patterns in the state of economic resilience within city groups and provides support for the developing theoretical system of "regional economic resilience" [22]. Additionally, it offers a theoretical basis and practical guidance for underdeveloped areas and old industrial regions worldwide to overcome regional challenges, and restore and improve their regional economic resilience.

## 2. Theoretical review

### 2.1 Theoretical review of relational networks

**Fig 1:**
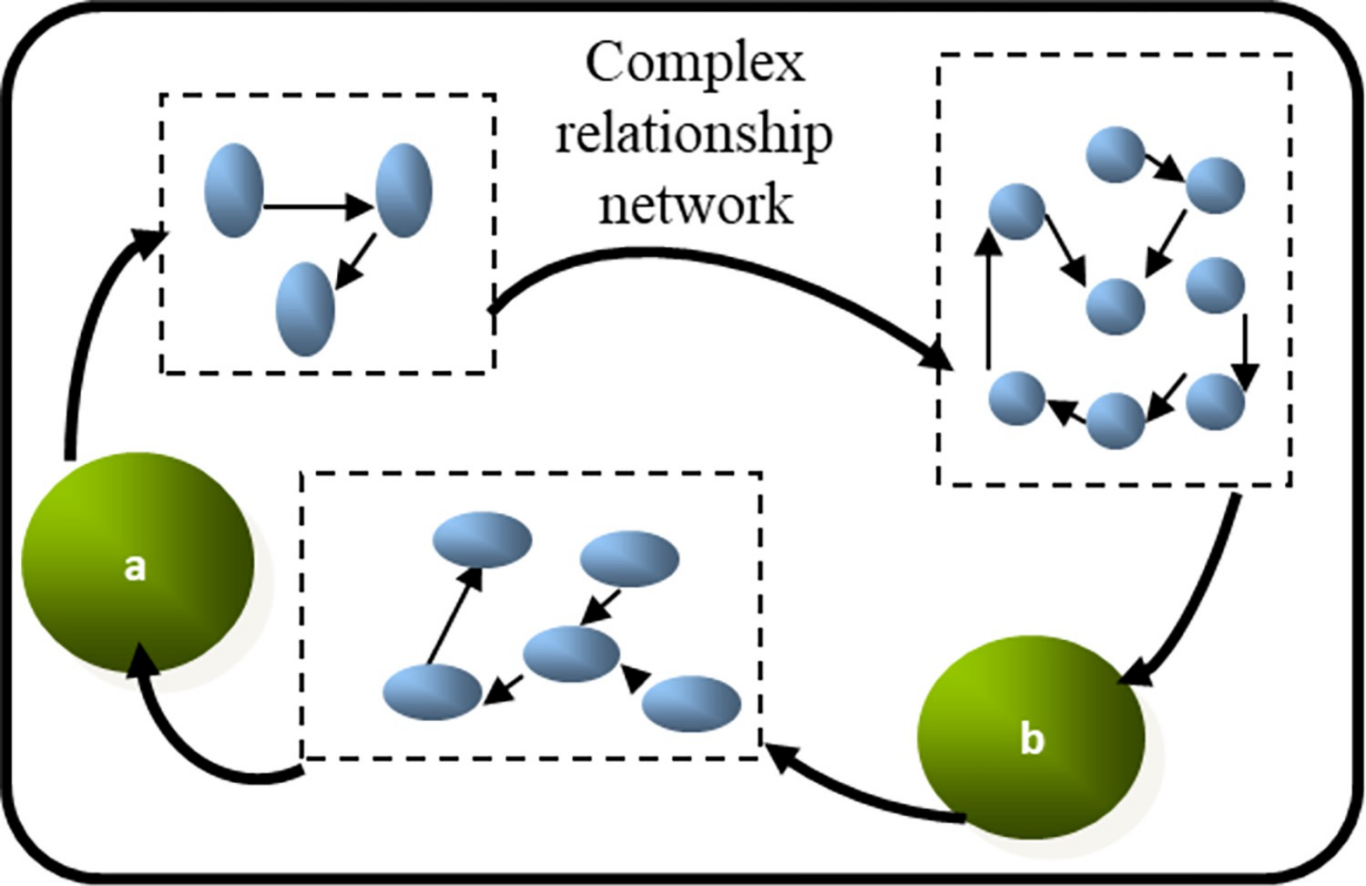
Nowak’s complex relational network structure.

The concept of the relational network originates from the field of sociology, where it pertains to the intricate system of social connections. This network is characterized as a dynamic system with multiple levels, structures, and orientations [17]. Scholars from other countries have previously applied this perspective to the study of inter-city relations. The notion of a "city network" was initially introduced by Dutch scholar Zonneveld, who argued that cities complement each other in terms of functions and subsequently generate economies of scale through transportation and information channels [18]. Since the mid-1990s, scholars like Smith [19], Derudder [20], Mahutga [21], and others have used air passenger transport data to investigate the global network, examining the relationships and degree of connectivity among world cities. Researchers such as Grubesic [22], Mitchelson [23], Malecki [24], and others have studied city interactions based on information flow. The Globalization and World Cities Research Network (GaWC) has described the world city network through the relationships between advanced producer service enterprises [25]. In China, empirical research on inter-city relations has become increasingly abundant since the 21st century. Studies vary in scale, with many focusing on the spatial organization of the urban system through the collection of urban relationship data. For instance, Jin & Wang 2005 [26], Xue 2008 [27], Wang et al. 2009 [28], and others have primarily examined the structure of the aviation network in China. Zhong & Lu 2011 [29], Feng et al. 2014 [30], and Wang et al. 2016 [31] explored the hierarchical structure of interregional cities by analyzing the connections between railway and highway passenger and freight flows. Chen et al.2015 [32] and Ye et al. 2015 [33] investigated the structural characteristics of urban networks within specific regions based on multiple traffic or information flows. Moreover, studies have emerged on the regional urban network structure from the perspectives of letter flow and enterprise production network [34–36]. With the advancements in internet technology, research on city-level networks based on GPS-tracked personal data has garnered significant attention, providing a fresh perspective on inter-city connections [37]. Other studies have examined inter-city relationships based on multiple traffic and information flows [38–40], suggesting that enhanced inter-city traffic information accessibility and higher levels of external connections contribute to the development of urban agglomerations. Despite the extensive research on inter-city relations in China, most studies based on communication network infrastructure have primarily focused on single-factor flow characteristics, while insufficient attention has been given to understanding how inter-city relationship networks form and their impact on the economic resilience of urban agglomerations. Hagedoorn et al. 2006 [41] argues that the inter-city relationship network possesses two crucial network capabilities: a central-based network capability and an efficiency-based network capability. The central-based network capability focuses on enhancing the strategic position of individuals within the network, while the efficiency-based network capability emphasizes the prompt and successful identification of cooperative partners necessary for division of labor. The complex and interactive relationships between cities form the external environment of cities [42]. The essence of individual network capability lies in the ability to coordinate and establish cooperative relationships with external networks, with the primary objective of gaining additional knowledge, information, and other resources. The study of urban network theory in urban systems indicates a shift in trends from attributes to relationships, from ranking to networking, and from closed systems to open systems [43]. Some scholars have examined the intricate structure of China’s inter-city network, drawing insights from economic and industrial relations [44], tourism flow [45], and transportation networks. However, due to challenges in data acquisition, research on inter-city networks remains limited. Crespo et al. 2014 [46] identified three types of inter-city relationship networks: random networks, core-edge networks, and resilient networks. While research on the first two network types is relatively advanced, understanding the formation of regional resilient networks is still in its early stages. A Chinese researcher studying international economic cooperation argued that people’s willingness to cooperate is shaped by their culture, knowledge, technology level, and openness to external influences [47]. These factors determine the structure of relationships among individuals and ultimately impact the stability of the entire cooperation system. Nowak et al. 2014 [48] further explored this topic by examining the concept of self-organization and uncovering the relationship dynamics influenced by individual willingness, particularly the intricate structural characteristics of the network (refer to Fig 1).

**Fig 2:**
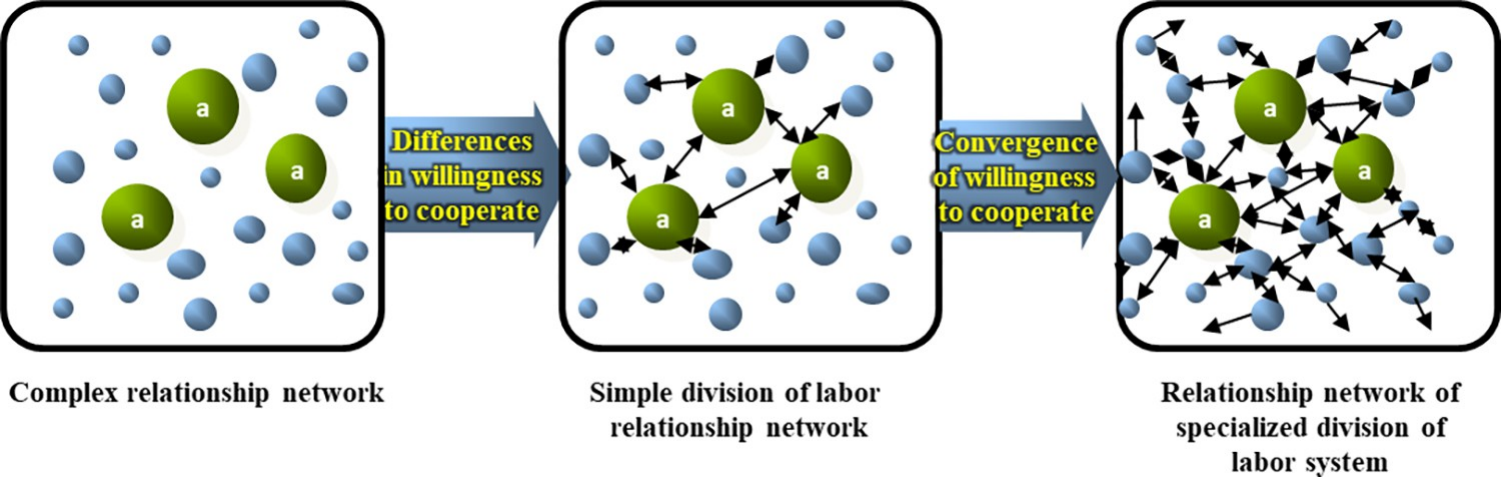
Relationship network type.

We discovered three key aspects in the study of regional economic cooperation and industry-university-research collaboration, regardless of whether they were on a micro or macro level (see Fig 2). The variation and nature of the relationship network among behavioral agents depend on their willingness to interact with one another. The inclination to engage with others is influenced by the inherent characteristics of the subject. When the subject’s inherent culture promotes closedness and a lack of willingness to cooperate, the relationship network takes on a competitive state. On the other hand, when the inherent culture is extroverted, the relationship network becomes a simple cooperative division. If the inherent culture fosters a convergence of willingness for open cooperation, the relationship network adopts a professional division system state, resulting in a diverse, flexible, and spillover-prone collaborative body.

### 2.2 Theoretical review of regional economic resilience

**Fig 3:**
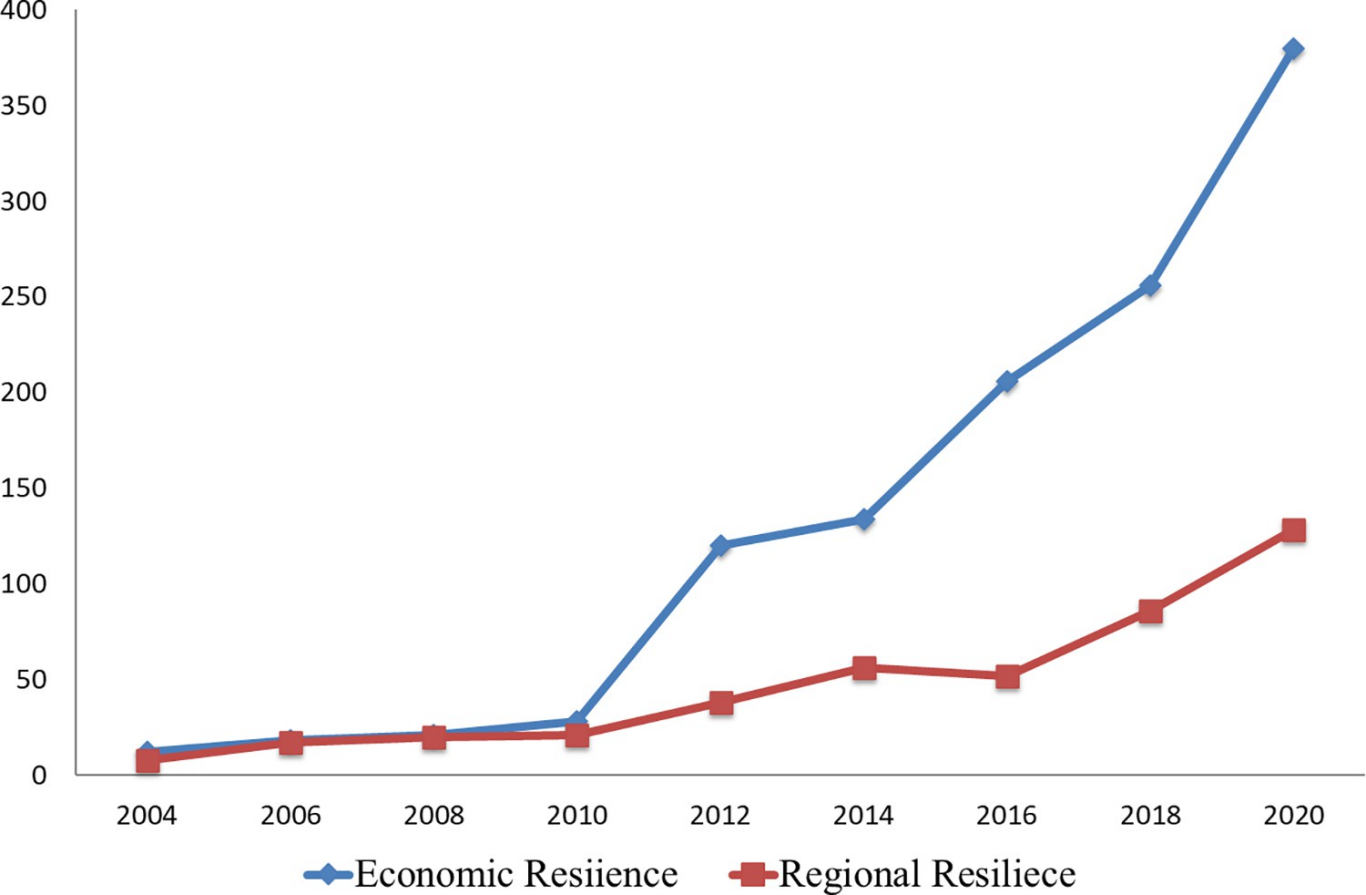
Literature statistics of regional resilience and economic resilience from 2004 to 2020. (Note: The literature is from EBSCO-Academic Search Premier).

The term "Resilience" has its roots in the Latin word "resilire" and finds its origins in various fields such as physics, engineering, and ecology. It encompasses the concept of a system’s ability to maintain stability and return to its original state after experiencing external disturbances. In recent times, resilience has gained attention in the realms of economics and geography. Economic resilience explores the variations in different regions’ capacity to withstand risks, recover from setbacks, and foster economic growth. It sheds light on the underlying causes of economic stagnation in older industrial areas. Currently, economic geography and regional economics research place significant emphasis on this topic (see Fig 3).

Studies on economic resilience primarily approach it from the perspectives of evolution theory and equilibrium theory. Evolution theory views resilience as the economic system’s ability to bounce back from external shocks, with different stages of regional development and evolution exerting varying degrees of influence on regional economic resilience [49]. Researchers such as Crespo et al. 2014 [46] have observed that resilient relational networks, which exhibit a high degree of correlation, tend to possess a tightly connected core (representing adaptability) and moderately loose connections at the edges (representing adaptability). Therefore, regional relational networks must strike a balance between strong associations with highly clustered nodes, ensuring efficiency, and weak associations with relatively discrete nodes that hold potential for external development.

**Fig 4:**
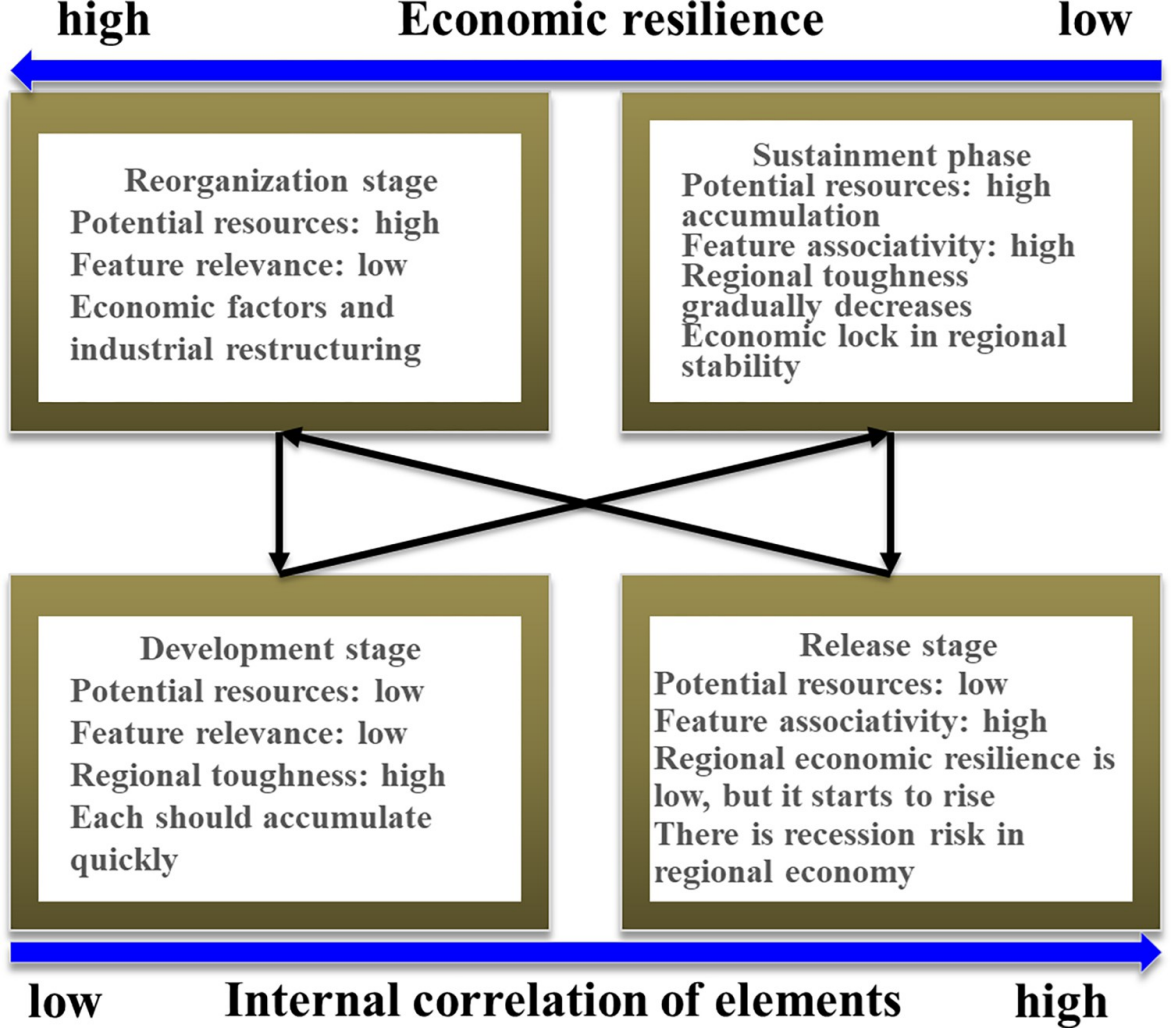
Regional economic adaptability model.

According to Chinese scholars, regional economic resilience encompasses qualities such as resilience, regeneration or reorganization, and innovation, which indicate the direction of diverse regional development [50]. Simmie and Martin [49], drawing inspiration from the complex adaptation scientific theory, incorporated the concepts of system-related degree and capital accumulation into the investigation of regional economic resilience. They proposed that the development of regional economic resilience within the adaptive cycle can be characterized by two adaptation phases: Firstly, the regional economy goes through a progression of emergence (restructuring stage)–development (opening stage)–stability (maintenance stage) with increasing rewards. Secondly, the regional economy also undergoes a decline or transformation process of rigidity (maintenance stage)–decline (release stage)–reconstruction(restructuring stage) (Fig 4).

Currently, there is limited discussion and detailed exploration of resilience studies grounded in evolution theory. China has recently seen the emergence of research fields such as innovation geography and evolutionary economic geography. Although Chinese scholars have acknowledged the development of innovation geography in the Western context [51], few have recognized the strong connection between this field and studies on regional resilience. Furthermore, Chinese scholars tend to focus on measuring regional resilience through case studies, particularly using quantitative approaches [52]. The proposed theoretical and technical framework for economic resilience can only account for the natural evolutionary process of gradual regional development. However, the mechanisms behind the formation of regional economic resilience at the network level, as well as the dynamic influences of interactions between different subjects on regional economic resilience, have not been adequately explained [53]. The theory of "resilience" broadens the approaches and frameworks used in studying regional economies and addresses the evolving demands and transformative aspects of regional development. It emphasizes the formation of interconnected networks between cities influenced by institutions, culture, customs, knowledge, and technology. These networks exhibit noticeable spillover effects across regions, playing a vital role in enhancing economic resilience and promoting recovery. Unfortunately, because there hasn’t been enough research to support this notion, the available literature mainly relies on case studies and lacks any useful quantitative analytic technique.

## 3. Quantitative expression of relationship network and regional economic resilience

### 3.1 Quantitative expression of relational network

Complex networks are pervasive in both human society and the natural world [54]. They provide an abstract description and visualization of various complex systems that are comprised of interacting relationships. Consequently, complex networks have emerged as crucial tools for investigating numerous phenomena in natural and social systems. In the context of inter-city relationships within an urban agglomeration, the interplay between cities, influenced by their distinctive cultures, gives rise to a complex network structure. This structure reflects the division of labor, the extent of mutual influence, the flow of resources between cities, and the resilience of each city to risks. The inter-city relationship network is shaped by factors such as cultural norms, knowledge and technology, and systemic policies. Consequently, the researchers in this study focus on examining the impact of changing cooperative intentions, which are indicative of a city’s underlying culture, on the knowledge spillover effect within the inter-city relationship network (measured by technological space distance) [55]. Additionally, they also explore the influence on market structure (represented by market concentration) as an expression of institutional effects within the relationship network, and the technological symbiosis index (indicating the interconnectivity of industries within the network, expressed by the degree of technical core relations) [56]. These analyses help determine the nature and spatiotemporal characteristics of the relationship network, ultimately shedding light on the resilience mechanisms of urban agglomerations.

#### (1) The expression model of knowledge spillover effect

Recently, there have been advancements in the study of the knowledge spillover model through research and empirical analysis conducted domestically and internationally. Experts in regional economics and economic geography share the belief that the knowledge spillover effect of clustered firms is not only influenced by physical proximity but also by the proximity of knowledge and technology. The extent of technological dissimilarity can be measured by the spatial distance between locations. Klaus developed models that incorporate both geographical and technological distances to examine knowledge spillover effects, which can be summarized as follows [57]:

$$SP_{i,k,l}=\sum_{l=1}^{n}\sum_{i\neq j}\left[(1-d_{i}^{geo})(1-d_{j}^{geo})\times \frac{1}{1+d_{k,l,t}^{teck}}\times G_{i,j,k,l,t}\times e^{\frac{G_{i,j,k,l,t}}{ac_{i,t}}}\right]+\sum_{l=1}^{n}\left[\frac{1}{1+d_{k,l,t}^{teck}}\times G_{i,j,k,l,t}\times e^{\frac{G_{i,j,k,l,t}}{ac_{i,t}}}\right]$$ (1)

Eq (1) indicates that at t time, enterprise i has the potential to experience a knowledge spillover effect associated with knowledge k. The variable$d_{i}^{geo}$represents how the technology distance of enterprise i influences knowledge spillovers; *ac*_*i*,*t*_ indicates the technology absorption capacity of enterprise I; *G*_*i*,*j*,*k*,*l*,*t*_ represents a disparity in knowledge stock;$d_{k,l,t}^{teck}$denotes the technical distance.

#### (2) Expression model of market structure

Market structure is represented by market concentration. The Herfindahl-Hirschman index, also known as the HI index, quantifies market concentration by adding up the market shares of all companies within a specific industry. Let us assume there are n companies operating in an industry, and we arrange them in descending order based on their market share. By assigning x_i_ to represent the sales of the i-th company and X to represent the total sales, we can use the index *Z*_*i*_ = *X*_*i*_/*X* to indicate the market share of each enterprise. HI index as:

$$HI=\overset{n}{\underset{i=1}{\sum }}Z_{i}^{2},0\leq HI\leq 1$$ (2)

#### (3) Expression model of technological symbiosis index

Western scholars utilize various quantitative indicators to assess the level of technological correlation. For instance, economic geographers make use of industry classifications within the national economy as readily available data to examine technical correlation. If two economic sectors fall under the same category, they are likely to share similar knowledge or skills, indicating a high level of technical correlation. Additionally, the movement of labor between sectors serves as a reliable indicator. If the amount of labor migration between two economic sectors significantly surpasses the standard benchmark for industries of similar size and growth, it suggests a high degree of technical correlation. Co-occurrence analysis, a commonly used technique among Western academia, comes in several forms as well. For instance, Neffke et al. 2013 [58] introduced the concept of Revealed Relatedness, which assumes that companies employing standardized machinery and individuals with similar knowledge backgrounds to produce similar products exhibit technological correlation. This correlation is measured through a symbiosis number(*L*_*ij*_).If companies from two distinct economic sectors employ individuals with comparable technical backgrounds to manufacture similar products, indicating a significant degree of correlation *L*_*ij*_, it is considered high when compared to the reference value of the symbiosis (*Q*_*ij*_) using:

$$RR_{ij}=k\frac{L_{ij}}{Q_{ij}}$$ (3)

Where: k is a statistical constant.

The formation and recovery of regional economic resilience are influenced by the interregional knowledge spillover effect, market structure, and technological symbiosis index. These factors also determine the process of change in regional economic resilience. The impact of these factors varies across different regions based on their respective capacities. Therefore, the relational network can be described as follows:

$$RN_{ij}(x_{j}-x_{i})=\sum_{j=1}^{N}\left[SP_{ij}(x_{j}-x_{i})+RR_{ij}(x_{j}-x_{i})\right]HI_{ij}(x_{j}-x_{i})$$ (4)

### 3.2 Quantitative expression of regional economic resilience

**Fig 5:**
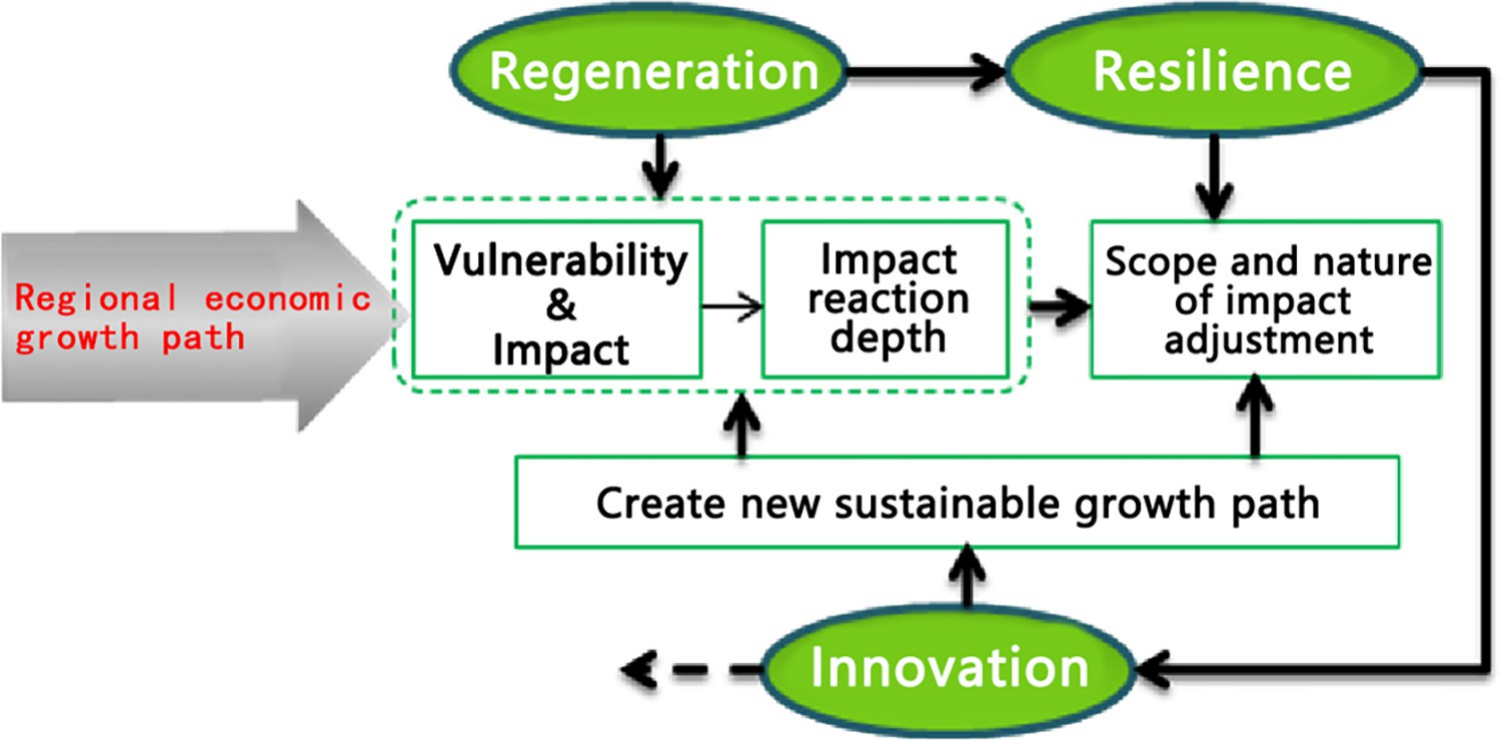
Regional innovation, resilience and regeneration.

The state of regional innovation, resilience, and regeneration on a multidimensional time-space scale reflects the economic resilience of a region (see Fig 5).

## 4. The relationship between the effect of network evolution of regional economic resilience model

**Fig 6:**
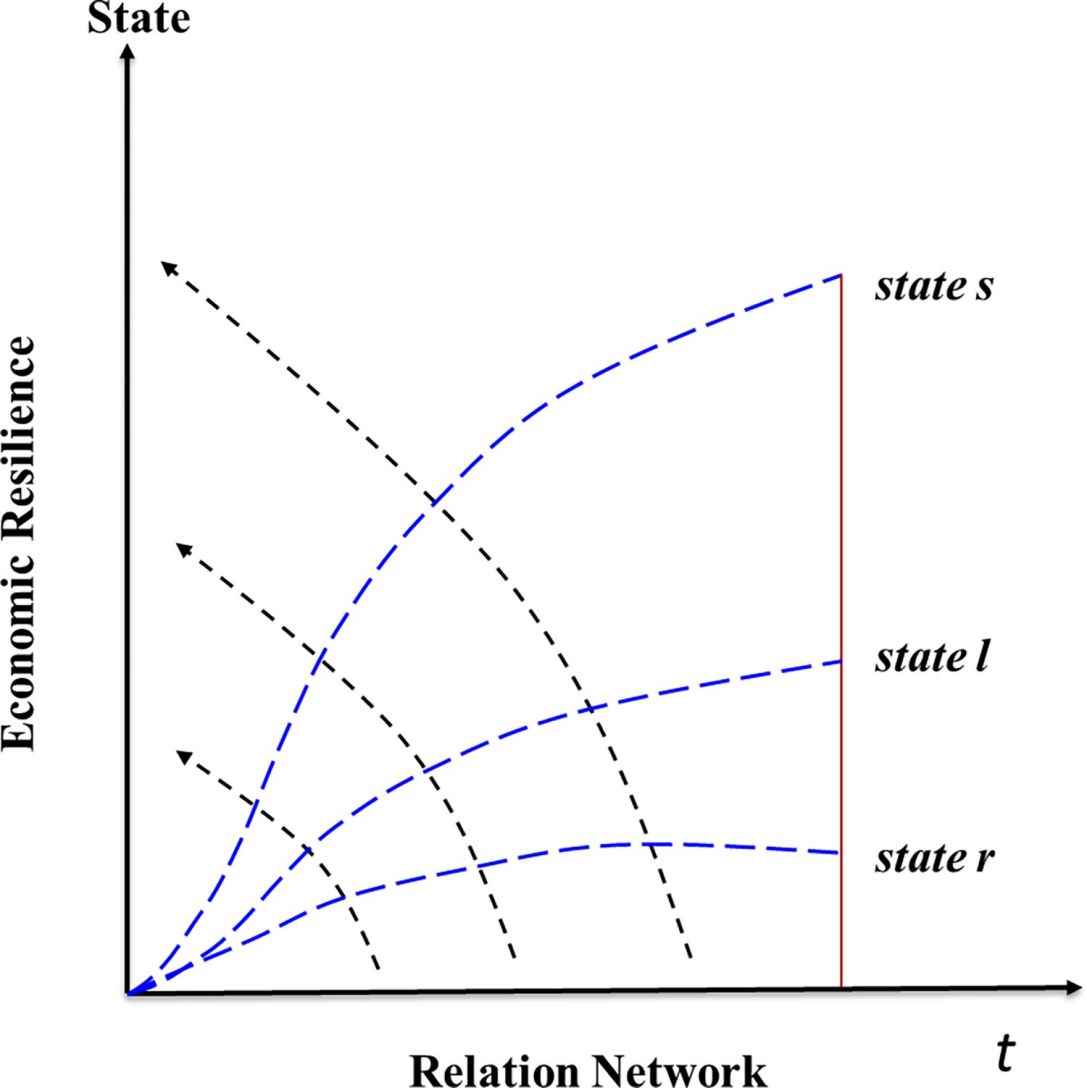
The logical frame of the research on the impact mechanism of relation network on city group economic resilience.

The central issue in studying the economic resilience of urban agglomerations is the transformation of inter-city relationships. This transformation is crucial for addressing the stagnation of regional economies. It is important to deeply analyze the influence of three key factors in the inter-city relationship network, namely the effects of knowledge spillover, the index of technology symbiosis, and the market structure, considering the cultural characteristics of the cities themselves. By examining the changes in the relationship network and their impact on economic resilience, we can understand the interconnected nature of the research mechanism, with innovation, adaptability, and recovery serving as indicators of economic resilience in urban agglomerations. To achieve this, we focus on the city relationship network as the starting point, study its impact process, evaluate its current state, and uncover the underlying laws that govern its effects. This forms the logical framework for our analysis (see Fig 6).

### 4.1 The process and law of the influence of urban cooperation intention on the relation network on city group

In the urban cluster comprising N individuals, the alteration in the desire to collaborate is represented by the willingness of each city along a linear scale and at a specific time t, as illustrated in the Table *x*_*i*_(*t*). As the transformation in each city’s readiness to cooperate is influenced by its inherent cultural values *G*(*x*_*i*_) and level of openness *Y*(*x*_*i*_), we can describe it as:

$$\frac{dx_{i}}{dt}=G(x_{i})+Y(x_{i})$$ (5)

The (initial) size of a city’s inherent culture can be attributed to its environment. When the desire to change primarily stems from other cities, the influence and interaction between cities vary. This distinction can be described as follows: *H*_*ij*_(*x*_*j*_−*x*_*i*_) If the influence strength of city j on city I is denoted as *k*_*ij*_, and the degree of acceptance of city I is denoted as *λ*_*ij*_, it can be expressed as *k*_*ij*_≥0,*k*_*ii*_ = 0.

The City Intend Evolutionary Dynamic Model for Cooperation:

$$\frac{dx_{i}}{dt}=(V(x_{i})+C(x_{i}))+\sum_{j=1}^{N}k_{ij}(Y(x_{j}-x_{i})-S(x_{j}-x_{i}))\exp(-\frac{{\left(x_{j}-x_{i}\right)}^{2}}{2\lambda_{i}^{2}})$$ (6)

We performed a correlation analysis to examine the level of cooperation between cities and the inter-city relationship network, and analyzed the factors and principles that affect the willingness to cooperate within this network. The "Möller and Svahn" [12] network capacity measure scale was utilized to analyze the impact of knowledge spillover effects in a network-based symbiosis model. This model considers the technology index, market structure, and a table of symbiosis to draw insights. Additionally, the study draws inspiration from the social judgment theory, which suggests that changes occur when critical differences diminish. The analysis follows “the first law of geography,” which highlights the correlation between space and establishes a correlation analysis model between urban extroverted degree, network, and three factors:

$$I=\frac{\sum_{i=1}^{n}(y_{i}-\bar{y})[\sum_{j=1}^{n}w_{ij}(y_{i}-\bar{y})]}{\sum_{i=1}^{n}{\left(y_{i}-\bar{y}\right)}^{2}}$$ (7)

**Fig 7:**
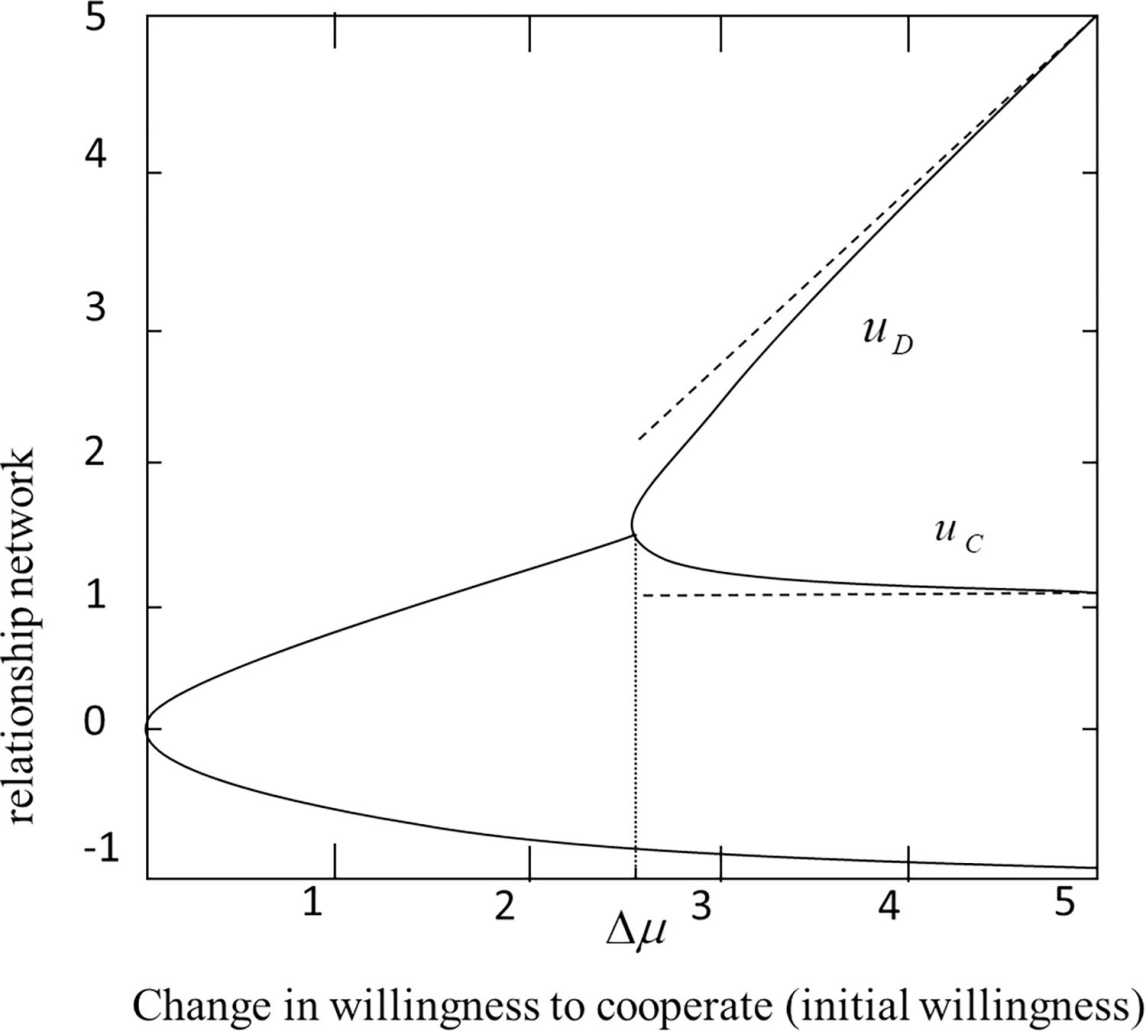
Change state of inter-city relationship network under the influence of cooperation intention (bifurcation state). Note: Fig 7 describes *y* = *f*(*u*) gradient curve, *γ* = 1,Δ*μ* = 3. In agreement of *u*_*c*_, deadlock situation *u*_*D*_, will disappear after *a* rising. There are exact solutions that approximate *u*_*c*_ and *u*_*D*_ (dotted line).

**Fig 8:**
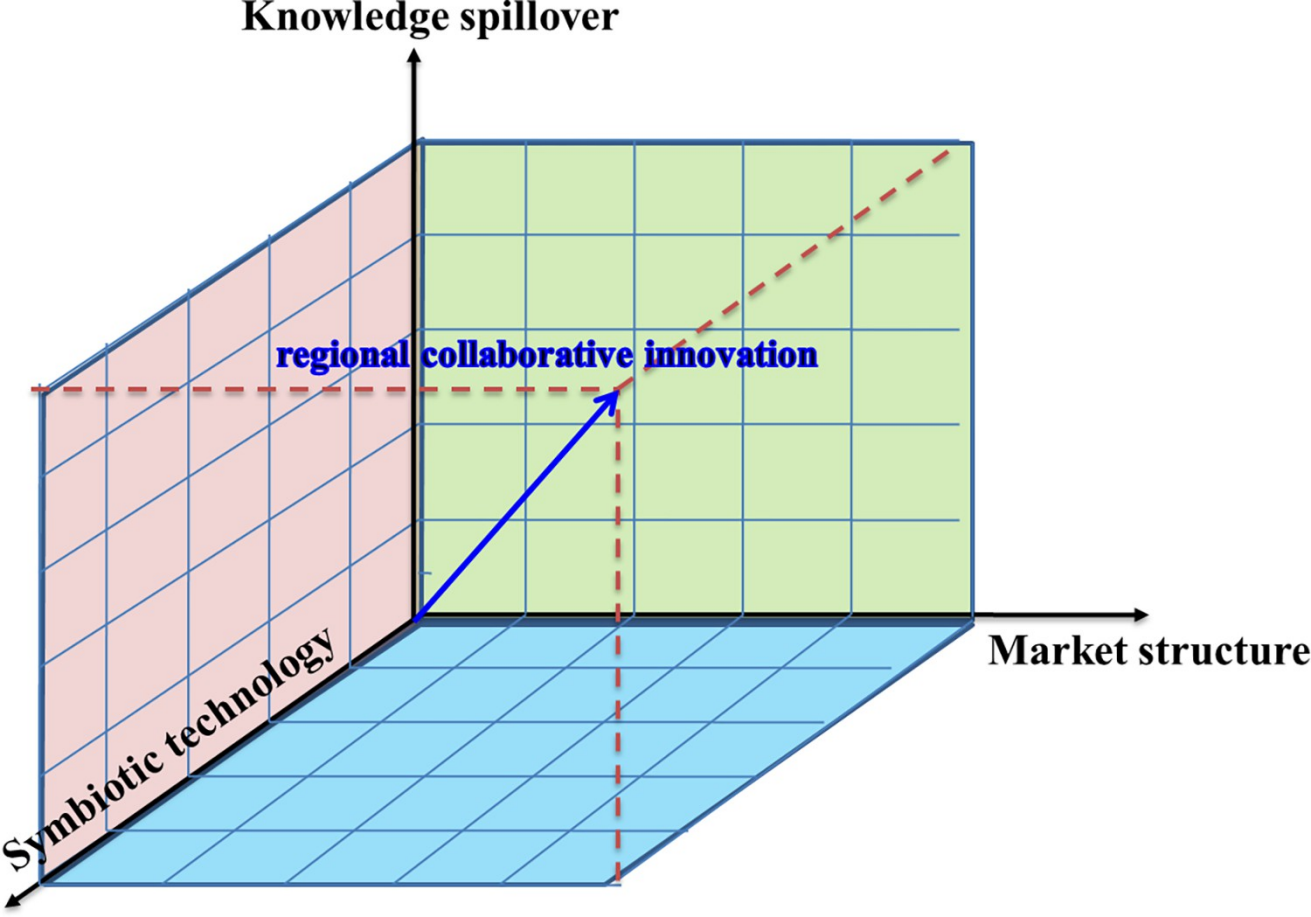
The relationship between relation network and economic resilience of city group.

Given that the inter-city relationship network is impacted by the willingness of cities to collaborate, the path of development for this network can split into two different directions, as depicted in Fig 7:

A three-dimensional model of the urban relationship network is constructed by considering the correlation between the knowledge spillover effect, market structure, and technology symbiosis index. This model is depicted in Fig 8.

Thus, by employing the principles and techniques of system dynamics, we can posit that the economic ability of urban agglomerations to withstand challenges relies on two distinct factors. First, there is a robust interplay of innovation, adaptability, and rejuvenation facilitated by the interconnectedness of relationship network, denoted as *Q*(*x*). Second, the waning influence of the city’s inherent culture (causing a decline in its external functionality) leads to significant disparities in cooperation among cities, thereby creating economic entrenchment resulting from the reversal of the relationship network which is denoted as *E*(*x*). This entrenchment directly affects the economic resilience of both the relationship network and the urban agglomeration, and their connection can be mathematically represented as a linear relationship:

$$\frac{dr}{dt}=\sum_{j=1}^{N}\left(Q_{ij}(x)+E_{ij}(x)\right)$$ (8)

The economic resilience of urban agglomerations is influenced by various factors within the inter-city network. When the economic resilience of these urban agglomerations reaches a specific level, connections will be formed between them, and the opposite is also true. To develop a theoretical model of how the relationship network impacts the economic resilience of urban agglomerations, social network theory and complex dynamics methods are utilized. The model can be summarized as follows:

$$S_{ij}(x_{j}-x_{i})=(x_{j}-x_{i}+\alpha_{ij})\exp(-\frac{{\left(x_{j}-x_{i}+\alpha_{ij}\right)}^{2}}{2\beta_{i}^{2}})$$ (9)

Where, *β*_*i*_ indicates the acceptance range of the influence of i on j, and *α*_*ij*_ indicates the degree of influence of i and j.

To create a search tree model with minimal complexity and hidden variables, we initially start with a basic model. During the search process, we gradually introduce new hidden variables and adjust the connections between them to increase their number. This leads us to obtain a Bayesian network (referred to as "Bayes net"). Within this network, the thickness of the edges connected to the leaf nodes indicates the degree of influence of the corresponding index. The Bayesian Information Criterion (BIC) is a shorthand term for this criterion.


$$\mathrm{BIC}(m|D)=\max_{\theta }\log P(D|m,\theta )-d(m)\log\frac{N}{2}$$ (10)

For simultaneous dynamic system, the theoretical equation of state is:

$$\{\begin{array}{c} \frac{dx}{dt}=x(e_{11}-e_{1})\\ \frac{dy}{dt}=y(e_{21}-e_{2})\\ \frac{dz}{dt}=z(e_{31}-e_{3}) \end{array}$$ (11)

The symbolic operation kit is used to determine the equilibrium solution, and the qualitative theory of differential equations is employed to assess the sign of the real part of this solution. The stability and stability conditions of the equilibrium solution are then evaluated. Furthermore, a numerical analysis is conducted to examine the state of the system described above.

### 4.2 Influence mechanism of relational network on resilience state of regional economy

Based on the previous research, the utilization of the Lorenz model and the logistic evolution model is employed to assess the trajectory of economic resilience within urban agglomerations influenced by inter-city relationship networks. This analysis aims to understand the progression from a state of weak economic resilience to a state of strong economic resilience, which is brought about by the inter-city relationship network. Additionally, the study examines the influence of three factors within the inter-city relationship network on the innovation, regeneration, and overall resilience of urban agglomeration’s economic resilience. Lorenz equation:

$$\begin{array}{l} x^{\prime }=-k(x+y),\\ y^{\prime }=-xz-y+rx,\\ z^{\prime }=xy-dz \end{array}$$

Application of relevant principle, can make *x*′ = *xu*(*x*), are Abel equation:

$$\frac{dv}{dx}=\frac{F(x)}{x}v^{n+1}+\frac{cx+b}{x}v^{n}$$ (12)

As the qualitative aspect of the relational network encompasses various categories, Eq (12) can be transformed into a logistic equation, and a multiple classification logistic regression approach is employed [59]. This model assesses the connection between the relational network and the economic resilience of urban agglomeration. Set m represents test variables Z_1_, Z_2_,……, Z_m_, where each variable Z_i_ contains a combination of P independent common factors F_1_,F_2,_…, F_p_(m≥p), and a unique factor U_i_(i = 1… m). The U_i_ factors are unrelated to each other, and F_j_ (j = 1… p) are also uncorrelated. Each test variable Z_i_ can be represented linearly by P common factors and its corresponding unique factor U_i_:

$$\left\{ \begin{array}{l} Z_{1}=a_{11}F_{1}+a_{12}F_{2}+\cdots +a_{1p}F_{p}+c_{1}U_{1}\\ Z_{2}=a_{12}F_{1}+a_{22}F_{2}+\cdots +a_{2p}F_{p}+c_{2}U_{2}\\ \ldots \ldots \ldots \ldots \\ Z_{m}=a_{m1}F_{1}+a_{m2}F_{2}+\cdots +a_{mp}F_{p}+c_{m}U_{m} \end{array}\right.$$ (13)

And satisfy:

- P≤m
- COV(F.U) = 0 (i.e., F is independent of U)
- E (F) = 0cov (F) = (^1^⋱_1_)_*p*×*p*_ = *I*_*p*_ namely the F_1_… were not associated with F_P_, and variance of 1, the mean to 0.
- E(U) = 0 COV(U) = Im U_1_,…,Um is irrelevant and is a standardized variable. Suppose z_1_…, z_m_ is also standardized, but not independent of each other.

For the values of two variables, $P(Y=1|X_{1},\ldots ,X_{p})=\pi$,$P(Y=0|X_{1},\ldots ,X_{p})=1-\pi$, logistic regression model is as follows:

$$\ln\frac{\pi }{1-\pi }=\log\mathrm{it}(\pi )=\beta_{0}+\beta_{1}X_{1}+L+\beta_{p}X_{p}$$ (14)

When there are *J*(*J*≥3) above, without order, the binary logistic regression can be extended for multiple logistic regression, classification is as follows:

$$\sum_{j=1}^{J}P(Y=j|X_{1},\ldots ,X_{p})=1,$$

$$\log\frac{P(Y=j|X_{1},\ldots ,X_{p})}{P(Y=J|X_{1},\ldots ,X_{p})}=\beta_{0}+\beta_{1}X_{1}+\ldots +\beta_{p}X_{p}$$ (15)

Upon solving, the solution *Z*_*ai*_ = *Z*_*bi*_ = … = *Z*_*ni*_ meets the conditions for economic lock-in of urban agglomerations.

The relationship between inter-city network (C) and the economic resilience of urban agglomeration (F) is:

$$R(\tau )=\int_{-\infty }^{+\infty }C^{*}F(t+\tau )dt|$$ (16)

Based on the research mentioned earlier, by employing the theory of self-organization and taking into account the characteristics and functioning of urban relationship networks as indicated in scheme (2), we can assert the following: a. Initially, cities exist as separate entities without any dependence on each other. b. The relational network among n cities exists, and there are no restrictions on the potential changes or variations within this inter-urban relational network. Then, ther Abel equation in the Lorenz model can be converted into:

$$b^{\prime }=(-k+\frac{g^{2}}{\gamma }D_{o})b-\frac{4g^{2}k}{\gamma \gamma_{11}}b^{*}bb+F(t)$$ (17)

The corresponding Fokker- Planck equation is:

$$f^{\prime }=[-\frac{\partial }{\partial b}(-ab-\beta {\left|b\right|}^{2}b)+c.c.+Q\frac{\partial^{2}}{\partial b\partial b^{*}}]f$$ (18)

Its potential function is:

$$\bar{V}(|b|)=-(-k+\frac{g^{2}}{\gamma }D_{o}){\left|b\right|}^{2}+\frac{2g^{2}k}{\gamma \gamma_{11}}{\left|b\right|}^{4}={\left|b\right|}^{2}(a+c{\left|b\right|}^{2})$$ (19)

Note: g^2^: according to the mechanism of urban agglomeration economic resilience by endogenous and exogenous constitute (mechanism); *γ* for urban agglomeration networks structure (structure has certain stability requirements); *k* for the city’s culture (the interaction between the city there was a block effect).

**Fig 9:**
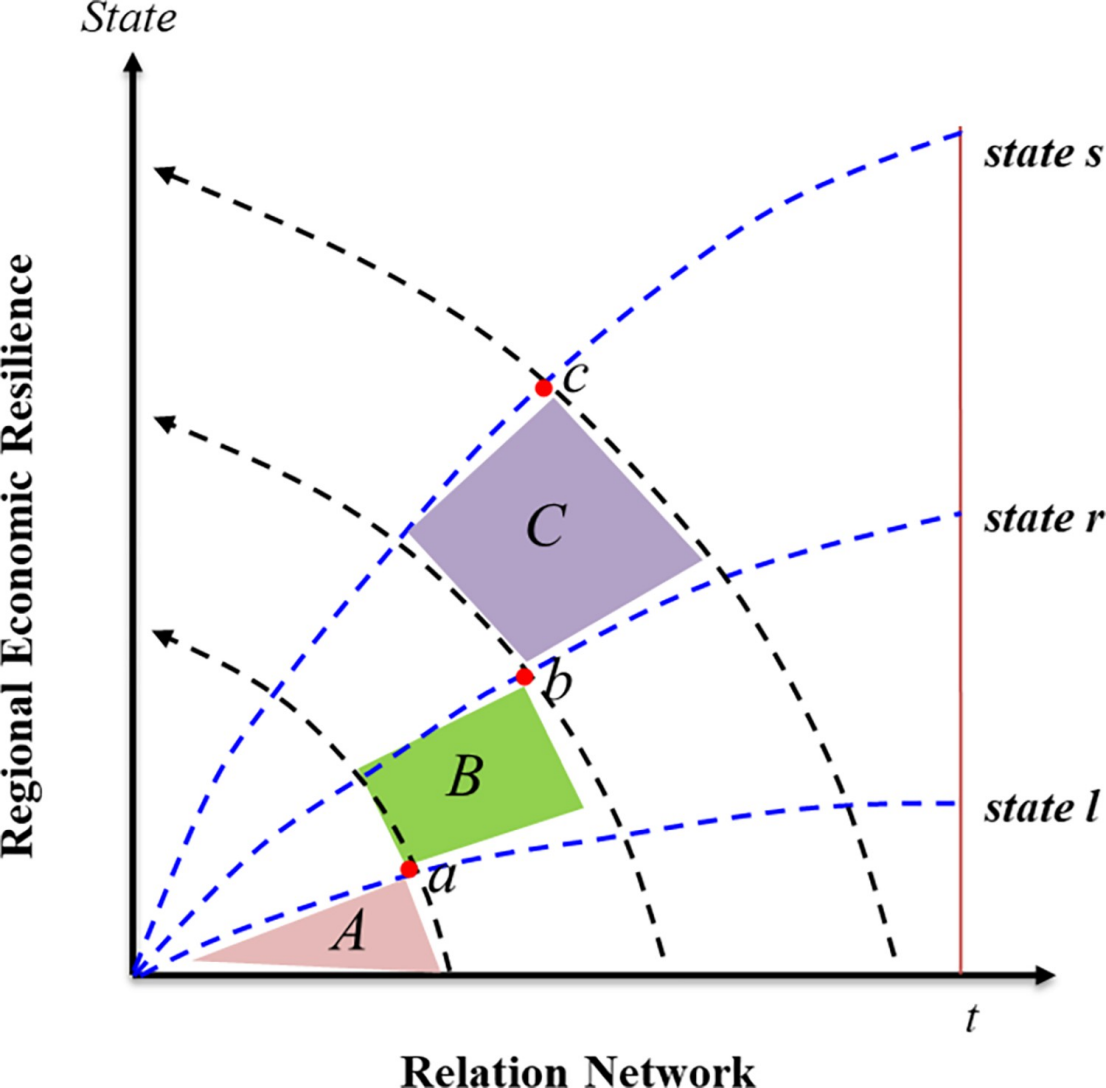
The impact mechanism of relation network on city group economic resilience state.

There was a significant shift observed in the system, when *a*/*c*<0, characterized by three crucial stable states representing the relationship between the urban network and the resilience of urban agglomerations’ economies. The first state when b^2^ = −*a*/*c* pertains to urban agglomerations without any economic resilience, while the second state *D*_*o*_>*kγ*/*g*^2^ signifies a critical transformation point where the economic resilience of urban agglomerations becomes constrained. Finally, the third state *D*_*o*_>*kγ*/*g*^2^ represents a threshold for yet another phase change, indicating a favorable condition for the economic resilience of urban agglomerations. Fig 9 illustrates these states, denoted as A, B, and C, which are critical thresholds for urban agglomerations’ economic resilience.

## 5. Discussion and prospects

The global economic turbulence and the progressive deterioration of the environment have heightened external risks to economic development. Examining regional economic resilience holds significant importance in enhancing economic stability. In this paper, we employ a complex network analysis method, focusing on inter-city relations, to construct an analytical framework called "city cooperation intention → inter-city relationship network → regional economic resilience". By incorporating principles from biological evolution and evolutionary economy, an evaluation system for inter-city relationship networks is established, investigating the influence of inherent city culture on these networks. Drawing on existing research findings, the study employs the structural force theory and three-dimensional coordinate method to analyze the relationship between the relationship network and regional economic resilience. Additionally, a mechanism model for the impact of the relationship network on regional economic resilience is established. The paper describes the trajectory of the "three factors" of the relationship network’s effect on the state of regional economic resilience, thereby enriching the research on regional economic resilience within the field of evolutionary economic geography.

Through a systematic review of relevant literature and in-depth theoretical discussions, we draw the following conclusions: (1) The inter-subjective relationship network presents three typical characteristics: competitive relationship network, simple collaborative division of labor relationship network and specialized division of labor professional network. The changes and types of relationship networks are determined by the subject’s willingness to interact with other subjects, and the subject’s inherent culture determines the change of willingness. Differentiated willingness to cooperate can promote the evolution of competitive relationship network to simple collaborative division of labor relationship network, and convergent willingness to cooperate can further promote the evolution of simple collaborative division of labor relationship network to specialized division of labor professional network. (2) The connotation of regional economic resilience has not yet formed a unified conclusion, and there is no effective analytical method for quantitative expression. Currently, it is recognized by most scholars that regional economic resilience is characterized by resilience, regeneration or reorganization, and innovation, and reflects the development trend of regional differences. Existing research mostly focuses on reflecting the relationship between regional economic resilience and other factors through case studies. However, there is still insufficient research on the impact of the inter-city relationship networks established by factors such as institutions, culture and customs, knowledge and technology and their spillover effects on the recovery and enhancement of economic resilience. (3) Complex systems theory is an effective tool for describing complex relational networks. The relationship network between cities can be established from knowledge spillover effect, market structure and technological symbiosis index, and its type and time-space characteristics can be analyzed. The quantitative expression of relationship network is the basis to reveal the mechanism of regional economic resilience. A three-dimensional coordinate method and structural force theory can be used to construct a quantitative expression of regional economic resilience. Innovation, resilience, and regeneration are the decisive element subsystems of regional economic resilience. Under the influence of dialectical unity, the logical relationship between the evolution of relationship networks and the mechanism of regional economic resilience is manifested as taking the urban relationship network as the entry point, the impact process as the research core, the state as the judgment basis, and the revelation of laws as the foothold.

**Fig 10:**
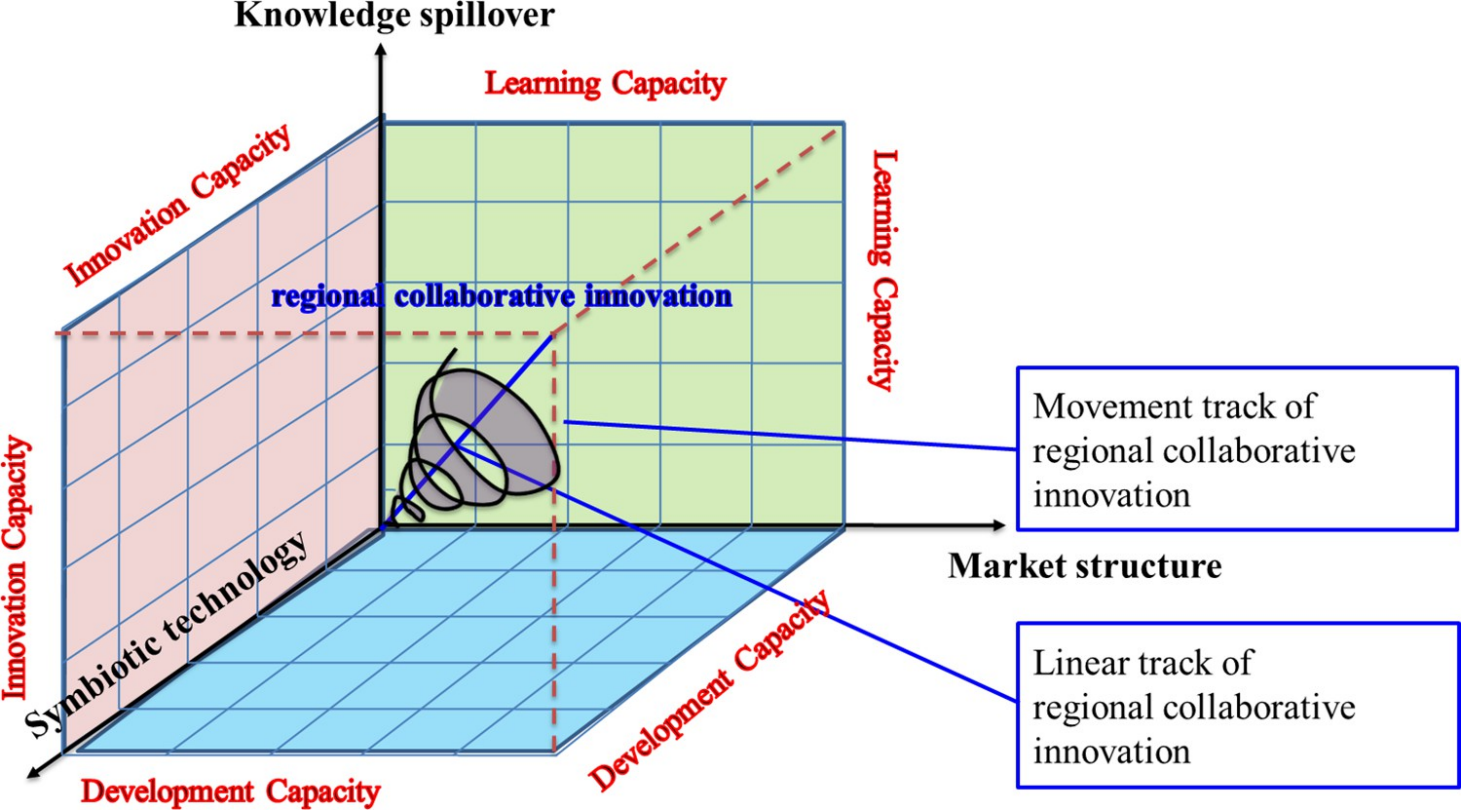
The frame theory of the impact mechanism of inter-city relation network on economic resilience of city group.

Additional investigation is warranted regarding the aspect of inter-city connections when it comes to regional economic resilience: (1) The establishment of inter-city relationship networks should pay more attention to policy impact. The inter-city relationship network refers to the way cities interact with each other, influenced by their respective cultures. It is characterized by a complex network structure that reflects the division of labor, the level of mutual influence, the flow of resources, and the ability of each city in an urban area to withstand risks. This network is shaped by factors such as culture and customs, knowledge, and technology, as well as systems and policies unique to each city. The inter-city relationship network encompasses the interactions and overall state of cities within an urban agglomeration, which impact the allocation of resources, innovation activities, and the division of labor and specialization within the urban area. This study does not consider the important role of institutions and policies in the establishment of inter-city networks as the main influencing factors. Therefore, when promoting urban development, it is crucial to focus on fostering inter-city relations through political policies. (2) Urban openness can be regarded as one of the crucial factors in establishing inter-city relationship networks. The analysis is based on the concepts of the knowledge spillover effect, technological symbiosis index, and market structure expression model of relational networks. By considering the spatial auto-correlation principle known as the "first law" of geography, we find that the evolution of inter-city relational networks follows a bifurcated trajectory. This means that changes in these networks are influenced by the willingness of cities to cooperate with each other. Additionally, the degree of openness of cities also affects their willingness to cooperate, thereby impacting the establishment of inter-city relationship networks. To enhance the formation of these networks, it is recommended to improve the openness of cities. However, it is important to note that this paper does not adequately discuss the potential negative impact of one-way outflow resulting from city openness. (3) Under ideal conditions, the impact of the three elements of the inter-city relationship network on regional economic resilience needs to be further clarified. This paper examines the linear process of how the three components of a relational network impact the innovation, resilience, and regeneration of economic resilience in urban areas. It focuses on the study of the three elements of inter-city relationship network and the role of the main factors affecting regional economic resilience as well as their quantitative expression and mechanism. In an optimal scenario, the economic resilience of urban agglomerations follows a spiral motion trajectory when influenced by these three elements (refer to Fig 10). This perspective holds theoretical importance in uncovering the evolutionary patterns of regional economic resilience and shaping regional economic policies. Moreover, it carries practical significance in enhancing the economic resilience of various regions amidst the ongoing global changes.

(4) The willingness of cities to cooperate and the network of relationships between cities can be incorporated into the regional economic resilience evolution system. Understanding how urban cooperation willingness affects the network of relationships between cities is crucial in uncovering the ways in which inter-city relationships contribute to the economic resilience of urban agglomerations. To investigate the dynamics between "urban cooperation willingness," "inter-city relationship network," and the "economic resilience of urban agglomerations," it is essential to comprehensively examine the analytical framework of relational economic geography and complex networks. By examining the willingness to cooperate and the relationship network among cities, it is possible to gain insights into the patterns that govern the regional economic resilience, treating it as an ongoing process. This paper focuses on the interaction paths between the three complex systems, and explores the specific relationship between the inter-city relationship network and urban economic resilience based on the willingness of cities to cooperate. It is still insufficient to integrate the willingness of cities to cooperate and the inter-city relationship network as the factors of action into the law of regional economic resilience movement.
